# Ontogeny and Polarization of Macrophages in Inflammation: Blood Monocytes Versus Tissue Macrophages

**DOI:** 10.3389/fimmu.2014.00683

**Published:** 2015-01-22

**Authors:** Adwitia Dey, Joselyn Allen, Pamela A. Hankey-Giblin

**Affiliations:** ^1^Department of Veterinary and Biomedical Sciences, The Pennsylvania State University, University Park, PA, USA; ^2^Graduate Program in Physiology, The Pennsylvania State University, University Park, PA, USA; ^3^Graduate Program in Immunology and Infectious Disease, The Pennsylvania State University, University Park, PA, USA

**Keywords:** tissue-resident macrophages, M1M2, microglia, adipose tissue macrophages, Kupffer cells, obesity, neurodegenerative disease, hepatic steatosis

## Abstract

The explosion of new information in recent years on the origin of macrophages in the steady-state and in the context of inflammation has opened up numerous new avenues of investigation and possibilities for therapeutic intervention. In contrast to the classical model of macrophage development, it is clear that tissue-resident macrophages can develop from yolk sac-derived erythro-myeloid progenitors, fetal liver progenitors, and bone marrow-derived monocytes. Under both homeostatic conditions and in response to pathophysiological insult, the contribution of these distinct sources of macrophages varies significantly between tissues. Furthermore, while all of these populations of macrophages appear to be capable of adopting the polarized M1/M2 phenotypes, their respective contribution to inflammation, resolution of inflammation, and tissue repair remains poorly understood and is likely to be tissue- and disease-dependent. A better understanding of the ontology and polarization capacity of macrophages in homeostasis and disease will be essential for the development of novel therapies that target the inherent plasticity of macrophages in the treatment of acute and chronic inflammatory disease.

Macrophages are a heterogeneous population of immune cells that have a range of roles in both the induction and resolution of inflammation. Tissue-resident macrophages promote tissue homeostasis and exhibit unique transcriptional profiles and characteristics depending on the tissue in which they reside (1). For instance, alveolar macrophages regulate pulmonary surfactant turnover while osteoclasts promote bone resorption, and red-pulp macrophages (RPMs) in the spleen promote red blood cell clearance and regulate iron recycling. In addition, multiple macrophage subtypes occur within a given tissue, and they can perform distinct functions depending on their anatomical location (2). For example, in the bone there are at least two types of tissue macrophages. TRAP^+^F4/80^−^ osteoclasts promote bone resorption while F4/80^+^CD169^+^TRAP^−^tissue-resident macrophages promote red blood cell development by providing a niche that promotes erythropoiesis and engulfing the extruded nuclei of red blood cell (RBC) progenitors at late stages of development (Figure 1A). Depletion of CD169^+^ macrophages results in impaired recovery of mice from hemolytic anemia (3). Recent studies suggest these two macrophage populations in the bone are replenished by distinct subsets of monocytes in response to stress. Mac3^+^F4/80^−^ monocytes regulate osteoclast activity but are not thought to be precursors of osteoclasts under homeostatic conditions (Figure 1A). However, these cells can differentiate into osteoclasts under inflammatory conditions. Alternatively, in the presence of increased levels of extracellular heme resulting from conditions of stress such as hemolytic anemia, a subpopulation of monocytes in the bone marrow and spleen develop into F4/80^+^CD11b^hi^ progenitors termed pre-RPMs and subsequently into RPMs and F4/80^+^VCAM^+^ bone marrow macrophages that resemble RPMs to re-establish iron homeostasis (Figure 1A) (4).

**Figure 1:**
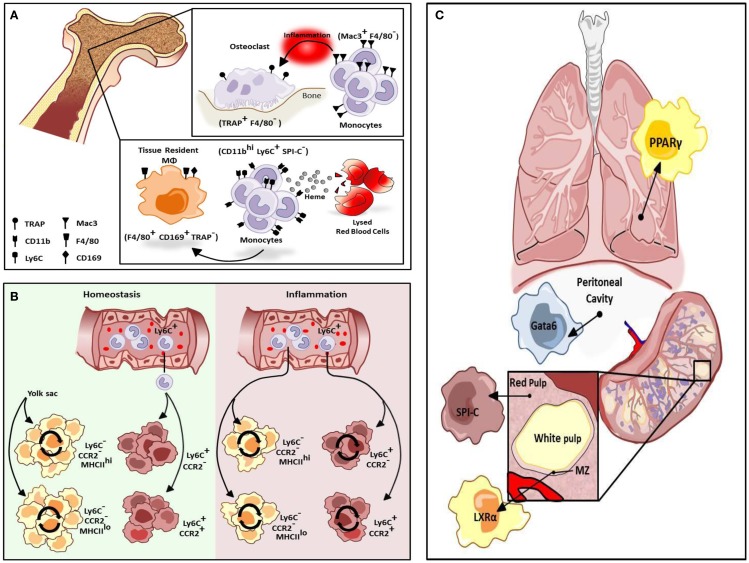
**Differentiation of resident macrophages in various physiological states**. **(A)** Two tissue macrophage populations reside in bone, F4/80^−^ TRAP^+^ osteoclasts and F4/80^+^ CD169^+^ TRAP^−^ tissue-resident macrophages (MΦs). Osteoclasts are developmentally dependent on Mac3^+^F4/80^−^ monocytes but only during inflammation. Alternatively, tissue-resident macrophages in bone can be maintained by a heme-induced subset of monocytes (CD11b^hi^ Ly6C^+^ SPI-C^−^). **(B)** Cardiac tissue-resident macrophage populations are primarily yolk sac-derived (YS) however a minor subset of the population is derived from fetal liver and HSC-derived progenitors. In homeostasis, primary yolk sac-derived cardiac resident macrophages are maintained through self-renewal but in response to cardiac insult, Ly6C^hi^ monocytes contribute to all four macrophage populations. **(C)** Selective transcriptional control plays an important role in the development of different types of macrophage populations. While red-pulp macrophages are dependent on SPI-C activity for development and maintenance, marginal zone (MZ) macrophage differentiation is mediated by LXRα. Alternatively, Gata6 is mandatory for the differentiation and proliferation of peritoneal macrophages while GM-CSF-dependent induction of PPARγ regulate alveolar macrophage development.

In the developing embryo, hematopoiesis begins in the yolk sac where primitive erythrocytes and macrophages develop in the absence of hematopoietic stem cells (HSCs). Subsequently, HSCs arise in the aorto-gonado-mesonephric (AGM) region and eventually migrate to the fetal liver where development of all hematopoietic lineages from HSCs occurs. In the neonate, hematopoiesis moves from the fetal liver to the bone marrow where it persists throughout adulthood. The traditional view of macrophage origin contended that tissue-resident macrophages develop from bone marrow-derived monocytes. It was subsequently believed that tissue-resident macrophages derive from Ly6C^−^ “resident” monocytes that traffic to tissues under homeostatic conditions. However, studies utilizing Runx1^CreER^ and Csf1^CreER^ mice, which allow for tamoxifen-inducible activation of Cre during the early stages of yolk sac hematopoiesis crossed with Rosa^26^ mice to label yolk sac-derived (YS) cells (5) have indicated that some tissue macrophages arise from the yolk sac and seed tissues in the embryo where they repopulate throughout adulthood (6, 7). Studies utilizing Flt3^Cre^ mice to mark HSC-derived macrophages confirm that YS tissue macrophages (Flt3^Cre−^) are distinct from HSC-derived macrophages (Flt3^Cre+^) thus challenging the traditional view of macrophage ontogeny. Kupffer cells, microglia, and cardiac tissue macrophages are primarily YS and are maintained throughout adulthood. These cells are independent of the transcription factor Myb, which is required for the development of HSCs, suggesting these populations of tissue-resident macrophages develop and persist independent of contribution from HSCs (8).

While Langerhans cells are first seeded by yolk sac macrophages, these cells are largely replaced by fetal liver-derived monocytes at later stages of embryonic development as demonstrated by adoptive transfer of fetal liver monocytes to host embryos (9). In addition, studies utilizing the CX_3_C chemokine receptor 1 (CX_3_CR1) promoter, which marks fetal-derived resident macrophage populations to drive GFP, Cre or a tomoxifen-inducible Cre together with adoptive transfer studies have demonstrated that peritoneal macrophages and alveolar macrophages are also largely fetal liver-derived. While cardiac tissue macrophages are also largely YS, two minor populations also display contribution from fetal liver and bone marrow progenitors (10–12). Other tissue-resident macrophages including those from spleen, pancreas, and kidney also exhibit mixed contribution from fetal and adult HSC-derived precursors (13), while intestinal macrophages appear to be entirely derived from circulating monocytes (14, 15). While these studies make a distinction between YS and fetal liver-derived macrophages, a recent report describes an erythro-myeloid progenitor (EMP) population that develops in the yolk sac and later migrates to the liver. These progenitors are distinct from HSC-derived hematopoiesis, suggesting that YS and fetal liver-derived tissue-resident macrophages have a common origin in the yolk sac (16).

Unlike previous assumptions that macrophages are terminally differentiated cells, YS macrophages appear to have stem cell-like properties in that they can proliferate and self-renew (9). In the lung, tissue-resident macrophages are maintained locally in the steady state. Moreover, following irradiation and bone marrow transplantation, residual lung-resident macrophages can re-establish normal homeostasis when the development of donor-derived macrophages is compromised (13). These cells expand in response to colony-stimulating factors, M-CSF and GM-CSF, in an interleukin 4 (IL-4)-independent manner. It is therefore likely that, following irradiation, the expansion of resident macrophages is delayed giving the monocyte-derived macrophages a developmental advantage. Alternatively, in response to helminth infection, Th2-dependent production of IL-4 promotes the proliferation of lung-resident macrophages (17) and this proliferation is independent of colony-stimulating factor-1 (CSF-1) (18). However, while Langerhans cells are embryonic-derived cells that exhibit hallmarks of self-maintenance, these cells are eventually replaced by circulating precursors following non-myeloablative transplantation (19). Similarly, YS cardiac resident macrophages are maintained through local proliferation, while monocyte-derived cells can replace all populations of resident cardiac macrophages following depletion or in response to damage (Figure 1B) (10, 20).

The development and maintenance of tissue-resident macrophages is also under tissue-selective transcriptional control. The transcription factor Gata6 is responsible for the transcriptome profile of resident peritoneal macrophages as well as for their proliferation under homeostatic conditions and in response to inflammation (Figure 1C) (21) and this programing is regulated reversibly by retinoic acid-induced Gata6 expression (22). Reflecting the fetal origin of peritoneal macrophages, retinoic acid induces expression of Gata6 in fetal-derived macrophages but not bone marrow-derived macrophages (BMDMs) due to epigenetic silencing of the Gata6 locus in BMDMs (22). Alternatively, the development of alveolar macrophages from fetal monocytes shortly after birth is regulated by the GM-CSF-dependent induction of peroxisome proliferator-activated receptor γ (PPARγ), resulting in a transcriptosome profile unique to alveolar macrophages (Figure 1C) (23). The development of splenic RPMs in response to excess heme requires the transcription factor SPI-C (Figure 1C) (4), however, marginal zone (MZ) macrophages develop normally in SPI-C knockout animals. MZ macrophages play a critical role in the recognition and uptake of blood-borne antigens. While the differentiation of RPMs requires SPI-C, the differentiation of both populations of MZ macrophages is under the regulatory control of the nuclear receptor, liver x receptor α (LXRα) (Figure 1C) (24), and LXRα-deficient mice exhibit abnormal responses to blood-borne antigens.

Unlike previous assumptions that Ly6C^−^ monocytes are precursors of tissue-resident macrophages, it has recently been shown that these monocytes do not infiltrate tissues but rather have a “patrolling” function in promoting endothelial integrity. Alternatively, Ly6C^+^ “inflammatory” monocytes are actively recruited to inflamed tissues by the chemokine, chemokine ligand 2 (CCL2) where they promote inflammation and pathogen clearance. Analogous populations of monocytes in humans have been described based on gene expression analysis and are classified as CD14^hi^CD16^−^, which are the primary subset in healthy individuals and resemble Ly6C^+^ murine monocytes while the minor CD14^lo^CD16^+^ population of human monocytes shares gene expression profiles with murine Ly6C^−^ monocytes (25). Furthermore, the CD14^lo^CD16^+^ population of human monocytes, like Ly6C^−^ monocytes, also play a role in patrolling the endothelium (26). Ly6C^−^ monocytes derive in the circulation from the Ly6C^+^ population and exhibit an extended half-life compared with Ly6C^+^ monocytes (9). Intriguingly, recent studies indicate that a subset of Ly6C^+^ monocytes can traffic to tissues under homeostatic conditions where they fail to differentiate into tissue-resident macrophages, rather they acquire antigen, which they carry to the draining lymph nodes (27).

Under non-inflammatory conditions, tissue-resident macrophages largely exhibit an M2 phenotype that promotes tissue homeostasis and repair. However, upon infection, M1 macrophage activation is induced by the engagement of pattern recognition receptors (PPRs) by pathogen-associated molecular patterns (PAMPs) such as lipopolysaccharide (LPS), in cooperation with interferon γ (IFNγ) (28). These cells produce cytotoxic oxygen and nitrogen radicals and pro-inflammatory cytokines resulting in increased microbicidal activity. M2 macrophage activation is induced by the Th2 chemokines IL-4 and interleukin-13 (IL-13) (29, 30). Alternatively, during the resolution of inflammation, the balance of macrophage activation toward an M2 phenotype occurs in order to promote clearance of debris, inhibit the production of inflammatory mediators, and restore tissue homeostasis. M2 macrophages produce anti-inflammatory cytokines and express endocytic receptors, and these cells promote the clearance of apoptotic cells, proliferation, and wound healing (28). However, the relative contribution and level of plasticity of tissue-resident versus monocyte-derived macrophage populations is only recently beginning to be elucidated.

The M1/M2 macrophage subsets are most commonly distinguished based on the catabolism of L-arginine. While classically activated macrophages express increased levels of inducible nitric oxide synthase (iNOS), which converts L-arginine to L-citrulline and nitric oxide, alternatively activated macrophages express arginase I (ArgI), which catabolizes L-arginine to L-ornithine, a precursor of polyamines and proline. While Th1 and Th2 cytokines promote M1 and M2 macrophage activation, respectively, these phenotypes are observed in T cell deficient mice suggesting that T helper cells are not required to drive macrophage polarization *in vivo* (29, 30). Conversely, M1 and M2 macrophages are capable of promoting Th1 and Th2 differentiation, respectively, suggesting these polarized macrophage phenotypes play an important role in driving the immune response to environmental insult (29). Recent attempts have been made to re-classify macrophage subpopulations in response to a range of stimuli and increasingly complex combination of markers (31). However, *in vivo*, macrophages are exposed simultaneously to a diverse array of signals, so it remains to be determined to what extent distinct subpopulations exist *in vivo*.

The origin and plasticity of macrophages in chronic inflammation remains poorly understood and is likely variable depending on the tissue and the underlying cause of inflammation. Tissue-resident macrophages as well as monocyte-derived macrophages appear to have the capability of adopting both M1 and M2 phenotypes, however, the relative contribution of these subsets of macrophages to the progression and resolution of chronic inflammation remains enigmatic. Here, we will review current progress in understanding the complex relationships between tissue-resident macrophages and infiltrating monocyte-derived macrophages in homeostasis and chronic inflammation and their contributions to M1/M2 polarized phenotypes in homeostasis and disease with a focus on chronic inflammation in the brain, adipose tissue, and liver.

## Microglia: Resident CNS Macrophages

Microglia, parenchymal tissue-resident macrophages in the central nervous system, plays a central role in mediating tissue homeostasis in health and disease (32). Fate mapping studies conducted independently by Ginhoux et al. and Prinz et al. demonstrated the embryonic origin of microglia from c-kit^+^ EMP prior to the formation of blood–brain barrier and vascularization of the embryo (7, 33, 34). The colony-stimulating-factor 1 receptor (CSF1R) and its ligand, CSF-1, play a central role in macrophage development and maintenance. However, while CSF1R is essential for the development and maintenance of microglia, mice deficient for CSF-1 exhibit only a partial reduction in microglial cells. Recent studies have identified an additional ligand for CSF1R, interleukin-34 (IL-34), which plays an essential role in the development and maintenance of microglia. While mice deficient for IL-34 exhibit significantly decreased numbers of microglia and Langerhans cells, the development of other tissue macrophage populations remain largely intact (35, 36). After birth, the microglia undergo massive expansion in response to CSF-1 and IL-34 in order to populate the developing nervous system. Cortical, optical, and spinal cord microgliogenesis is sustained by the transcription factors Pu.1 and interferon regulatory factor-8 (IRF-8), and is independent of Myb, which is required for the development of hematopoietic stem cells (34, 37). Microglia are sustained and self-renew within host tissues throughout adulthood independent of progenitors from the bone marrow (38).

In a healthy brain, microglia are dispersed relatively uniformly throughout the parenchymal tissue. Microglia are characterized by morphological features that reflect their functional capacity. In a healthy brain, microglia are in a quiescent state or have a “down-regulated” phenotype exhibited by a ramified shape, similar to Langerhans cells that have short fine processes and thus increased surface area for tissue surveillance (39). This down-regulated phenotype is characterized by an attenuated innate immune function correlated with decreased expression of CD45, MHCII, and Fc receptors (39–41). Initially, it was believed that steady-state microglia are static, however, recent studies have characterized the quiescent phenotype as a more dynamic state in which microglia are performing housekeeping functions by constitutively surveying the parenchymal tissue (42). This dynamic state is characterized by increased expression of oxidative genes and favors homeostatic tissue remodeling and steady-state wound healing (43).

**Figure 2:**
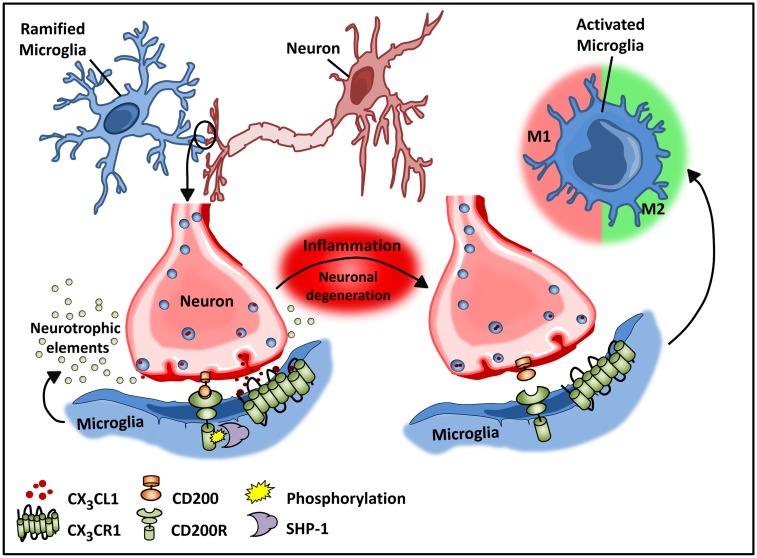
**Microglial–neuronal interactions in health and disease**. Healthy neurons expressing chemokine fractalkine (CX_3_CL1) and CD200 membrane proteins intimately interact with their respective transmembrane protein receptors on microglia, CX_3_CR1, and CD200R to sustain a down-regulated microglial phenotype. Microglial receptors have immunoreceptor tyrosine-based inhibitory motifs (ITIMs), which upon ligand–receptor activation suppresses downstream immune signaling through the recruitment of phosphatases including SHP-1. Chronic inflammation disrupts this intimate neuronal–glial interaction, thus releasing the microglial cells from a down-regulated inhibited state to an activated phenotype.

Recent studies by Butofsky et al. (44) demonstrate that resident microglia exhibit a distinct expression profile that is not observed in microglial cell lines and is distinct from M1 or M2 polarized microglia, but rather includes genes associated with nervous system development. The induction of this unique profile is highly dependent on transforming growth factor β (TGF-β) – *in vitro* in human microglial cultures, and these cells are absent in TGF-β knockout mice. The down-regulated phenotype of microglia is critical for normal neuronal growth, and intimate interactions between neurons and microglia are important for optimal synaptic growth and maintenance (Figure 2) (45). The interaction between neurons and microglia is fostered by the chemokine CX_3_C chemokine ligand 1 (CX_3_CL1; also known as fractalkine) and CD200 membrane proteins expressed on healthy neurons that interact with their respective transmembrane protein receptors on microglia, CX_3_CR1, and CD200R, respectively (32, 46–48). These microglial receptors carry immunoreceptor tyrosine-based inhibitory motifs (ITIMs) such that, upon ligand–receptor interaction, the ITIM based receptors suppress downstream immune signaling through the recruitment of Src homology 2 domain-containing phosphatase 1 (SHP-1) (32, 48–50). These cell–cell mediated interactions act synergistically with constitutively released neurotropic elements in order to create an environment fostering the down-regulated phenotype of microglia.

Whether or not bone marrow-derived cells can contribute to the microglial population remains under debate (reviewed in Prinz et al.). Bone marrow transplantation of irradiated recipient mice demonstrated that macrophages in the perivascular space and choroid plexus can be repopulated by donor cells (51, 52). While microglia are largely radio-resistant cells, upon irradiation and bone marrow transplantation, bone marrow-derived progenitor cells that are distinct from monocytes have been shown to seed the brain and differentiate into microglial-like macrophage cells (45). However, it is not clear whether BMDMs maintain the range of functions exhibited by YS microglia. In addition, irradiation results in the breakdown of the blood–brain barrier, thus whether the seeding of the brain with peripheral bone marrow-derived cells can occur under steady-state conditions in the presence of an intact blood–brain barrier is not known.

Like macrophages in the periphery, *in vitro* studies demonstrate that M1 and M2 activation can be induced in microglia by LPS and IL-4, respectively (40, 53). However, whether this phenotypic switching of resident microglia occurs *in vivo* remains unclear. Experimental models of Alzheimer’s disease (AD), aging, and multiple sclerosis (MS) (54, 55) have demonstrated microglial heterogeneity analogous to systemic tissue-resident populations. These studies suggest that early in disease progression, microglial cells develop an altered inducible “activated” state that is functionally different from steady-state microglia. This activated state is then further subdivided into a classical M1 and alternative M2 state (32, 40). Morphologically, activated microglia exhibit an amoeboid shape in contrast to the quiescent ramified shape of steady-state microglia (42). While the quiescent state of microglia is maintained by neuronal–glial interactions, in the course of inflammation, neuronal, and glial interactions are disrupted due to degeneration of neurons (43, 56). Disruption of CX_3_CL1–CX_3_CR1 interactions releases microglia from tonic inhibition (48), resulting in microglial activation as a consequence (Figure 2). Additionally, a unique “primed” microglial state, which exhibits a functional state somewhat distinct from the activated states, has been described (57). These microglia are thought to function as more of an adaptive response (57). This primed state refers to a mode of preconditioning underlying chronic inflammation whereby microglial cells are activated continuously by being persistently exposed to a pro-inflammatory milieu (32, 57). Importantly this priming state enables the conditioned microglia to mount a heightened response to secondary or future immunological insult in the course of disease progression. Primed microglia exhibit a more robust inflammatory response as compared to those that were not subjected to prior stimulatory challenges (32, 57). Additionally, the heightened inflammatory response to a physiological challenge is believed to promote a switch from a more anti-inflammatory to a pro-inflammatory reactive phenotype resulting in cellular stress and exacerbated inflammation (57).

Investigations in spinal cord injury (SCI), AD, and the animal model of MS, also known as experimental autoimmune encephalitis (EAE) demonstrate an increase in both M1 and M2 phenotypes, possibly acting synergistically in an attempt to mitigate inflammation (40). While both M1 and M2 transcripts increase in a majority of chronic CNS disease models, the M1 phenotype offsets the M2 phenotype in the early stages of chronic inflammation, disrupting normal neuronal/glial cross-talk, and promoting a pro-inflammatory milieu implicated in the progression of disease (39, 40, 53). With progressive degeneration, the M1 phenotype predominates, as M2 microglia exhibit decreased responsiveness to anti-inflammatory cues (53). Alternatively, the classically activated M1 phenotype is predominately induced by acute pathological conditions such as stroke, traumatic brain injury, or experimentally induced systemic inflammation by LPS (42). The M1 state in chronic and acute manifestations is characterized by the up-regulation of MHCII, CD86, and Fc receptors and increased production of pro-inflammatory cytokines such as interleukin-6 (IL-6), interleukin-1 (IL-1), and tumor necrosis factor alpha (TNFα) (58). Furthermore aging models hint at a possibility of phenotypic switch in microglial activation from an M2 to M1 state over time, coincident with decreasing neurogenesis and a growing inflammatory niche (32). However, it still remains unclear whether these populations of macrophages are due to phenotypic switching of resident microglia, or the activation states of infiltrating monocyte-derived macrophages. Interestingly, research in microglial senescence sheds light into the notion that microglial degradation precedes neurodegeneration and perhaps neurodegeneration is a secondary factor to aging induced dysregulation of microglial populations (59, 60).

Several studies of chronic inflammation in the CNS have demonstrated a degree of immune infiltration into the CNS, which is presumed to promote tissue remodeling as an attempt to maintain homeostasis (61, 62). The observed monocytic infiltration is attributed to the breakdown and weakening of the blood–brain barrier, which then facilitates the entrance of immune cells (63, 64). However, while monocyte infiltration has traditionally been considered a late event in disease development recent studies by Yamasaki et al. using an EAE model, demonstrated that infiltration of Ly6C^+^ monocytes occurs at the onset of disease and that these infiltrating cells are responsible for initiating nodal demyelination (65). It was further demonstrated that, while infiltrating monocytes are responsible for initiating nodal demyelination, resident microglia are relatively quiescent upon disease onset (65–67). The infiltrating monocytes promote disease progression though they do not appear to contribute to the resident microglial population (68). Similarly in a model of AD immune cell infiltration and localization of microglial populations in areas of amyloid β (Aβ) plaque deposits has been observed (69, 70).

Recently, researchers have been targeting microglial genes, such as TGF-β activated kinase (TAK1) to elucidate how tissue-resident macrophages are behaving during inflammation. While infiltrating monocytes are highly phagocytic and inflammatory, resident microglia are relatively quiescent upon disease onset. However, a recent study by Goldmann et al. demonstrated an essential role of microglia in the onset and progression of disease in an EAE model (71). By targeting TAK1 in microglia, but not peripheral monocytes/macrophages, they found that the lack of TAK1 in microglia inhibited the progression of inflammation in the CNS, highlighting the contribution of both resident microglia and bone marrow-derived monocytes in the onset and progression of EAE. However, in a model of EAE, infiltrating monocytes are not maintained and do not appear to contribute to the resident microglial population (68). Furthermore, recent studies using intravital correlated microscopy in zebrafish show that infiltrating phagocytes undergo cell death and are engulfed by resident microglia during resolution of inflammation (72). Popovich et al. investigated the potential for targeting CX_3_CR1 in a model of SCI as the ligand–receptor interactions between CX_3_CR1 and CX_3_CL1 are fundamental for optimal microglial function (47). Furthermore, Donelly et al. presented two important findings, targeted CX_3_CR1 deficiency/blocking in SCI results in enhanced recovery and decreased monocytic infiltration to injury site (48).

## Adipose Tissue Macrophages

**Figure 3:**
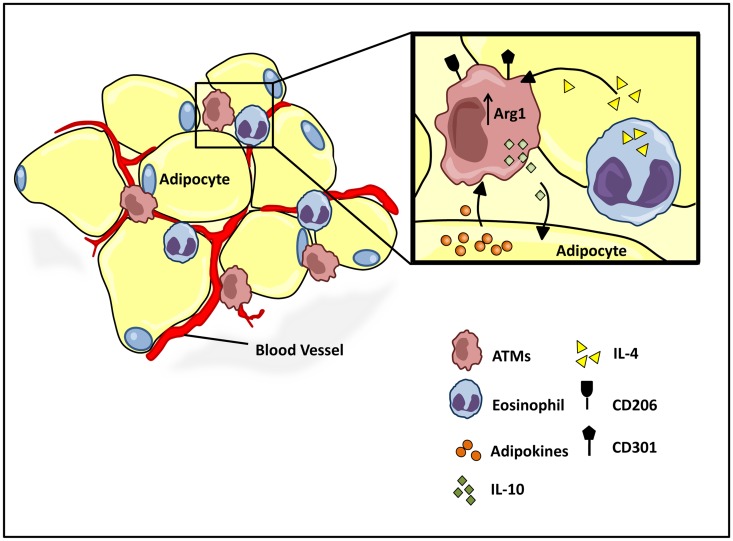
**Homeostatic regulation of ATM microenvironment**. Healthy adipose tissue contains a relatively low and uniformly dispersed population of alternatively activated M2 macrophages, expressing cell surface antigens CD206 and CD301. The M2 polarized state is maintained by eosinophil and adipocyte derived adipokine secretions, IL-4, and adiponectin, respectively. M2 ATMs maintain a homeostatic adipose milieu with IL-10 secretions, which in turn regulate glucose homeostasis within systemic tissues.

While it has long been clear that undernutrition can impair immunocompetence, more recently, the progression of obesity has also been attributed to a shift in immune function from an M2 to M1 type responses (73). Work by both Spiegelman (74, 75) and Lumeng et al. demonstrate critical interactions between adipose tissue metabolism and the immune system, and the active role of adipose tissue macrophage (ATM) polarization in the progression of obesity. Lean individuals in a non-inflammatory state maintain a relatively low percentage (~10–15%) of resident ATMs (76). Healthy adipose tissue consists of uniformly distributed alternatively activated M2 macrophages, expressing Arg1 and the cell surface antigens CD206 and CD301 (Figure 3) (77–79). The M2 polarized state of the ATM population in healthy adipose tissue is maintained by eosinophils (80), which secrete IL-4 (Figure 3) (81, 82). Polarized M2 ATMs secrete interleukin-10 (IL-10), which regulates glucose homeostasis within adipose tissue and systemic tissues including muscle (Figure 3) (83). In turn adipose resident invariant natural killer T cells (iNKT), expressing nuclear factor interleukin 3 regulated (E4BP4), induces the M2 phenotype through secretion of IL-10 (84). Healthy adipocytes, in turn, secrete anti-inflammatory adipokines such as adiponectin, a hormone that acts synergistically with IL-4 to exert anti-inflammatory effects through the activation of AMP kinase (AMPK) and signal transducer and activator of transcription 6 (STAT6), respectively (78, 85, 86). The alternative M2 state is further sustained by the transcriptional regulators peroxisome proliferator-activated receptor-δ (PPARδ), PPARγ, and Krüppel-like factor 4 (KLF4) (82, 87–89).

The intimate interaction between adipocytes and ATMs may reflect a common origin of these two cell types. Studies have shown not only cross-talk between ATMs and adipocytes, but a great deal of plasticity between these two lineages compartments (90, 91). Furthermore, while still controversial (92) several studies have suggested that some adipocytes and adipose progenitors may be hematopoietic stem cell-derived (93, 94). In addition, Coussin et al. have shown that hematopoietic stem cells that can reconstitute lethally irradiated recipient mice exist within adipose tissue (95), and more recent studies have supported the concept that adipose tissue is an extramedullary source of hematopoietic stem/progenitor cells (96, 97). Yet other studies have demonstrated the phagocytic capacity of pre-adipocytes and adipocytes suggesting they can adopt macrophage-like functions (85, 98). Interestingly, a recent study by Eto et al. has identified a novel subpopulation of ATMs present in perivascular regions of adipose tissue (99). This population of ATMs is characterized by the expression of CD206 and the stem cell marker CD34. The investigators further demonstrated that these cells can differentiate into the adipogenic lineage *in vitro*, suggesting the possibility that this subpopulation of ATMs might be a source of adipocytes *in vivo*. Whether these cells are hematopoietic-derived or whether they are precursors of the more abundant CD206^+^CD34^−^ ATMs is unclear.

In the late 2000s, Lumeng et al. identified adipose tissue inflammation as an important early event in the development of obesity related complications (82). At the onset of environmental and metabolic perturbation, such as chronic exposure to a high-fat diet (HFD), adipose tissue undergoes vast biochemical and morphological remodeling in part facilitated by infiltrating monocytes that then differentiate into ATMs. An excess of nutrients stimulates adipose tissue hyperplasia and hypertrophy as individual adipocytes enlarge with excess triglycerides (TAGs) and simultaneously undergo heightened levels of cellular stress imparted by endoplasmic reticular (ER) stress, hypoxia, release of excess free fatty acids (FFAs), increased reactive oxygen species (ROS) production, and adipocyte necrosis (77, 82, 100). The “stressed adipocytes” create an environmental milieu that promotes a more pro-inflammatory state exemplified with an increase in local IL-6, TNFα, and IL-1 production (101). Early adipocyte death is facilitated by ATMs in an effort to maintain homeostasis, followed by the development of a new range of pre-adipocytes (77, 102). Further studies demonstrated that adipocyte necrosis is not only an early event in diet-induced obesity (DIO), but it is a pivotal event, which results in the release of chemokines that attract circulating monocytes to the site of inflammation (101, 103). It is well established that throughout the course of adipose tissue inflammation, the resident macrophage population increases from ~10 to 50–60% associated with a parallel increase in F4/80^+^CD11c^+^ inflammatory macrophages (78, 82). Interestingly, Kosteli et al. demonstrated that weight loss also leads to a rapid recruitment and accumulation of ATM’s in white adipose tissue (WAT) (104). ATM accumulation occurs with lipolysis-induced release of excessive FFAs and subsequent clearance of lipolysis byproducts by WAT macrophages. Contrary to DIO ATM accumulation, weight loss induced ATM recruitment does not exacerbate the existing inflammatory state and ATM populations diminish with the declining rate of lipolysis (105).

At the onset of a physiological imbalance in the adipose niche, such as weight gain, the uniformly dispersed small population of resident macrophages exhibits an alternatively activated CD206^+^CD301^+^Arg1^+^ M2 phenotype (79, 82, 87). The ensuing pro-inflammatory milieu created by stressed adipocytes and necrosis attracts circulating monocytes to the site of inflammation as early as 10 weeks from the onset of HFD introduction. Circulating Ly6C^+^/chemokine receptor-2 (CCR2)^+^ monocytes are recruited and differentiate into the classically activated CD11c^+^CD11b^+^F4/80^+^ M1 ATMs expressing high levels of iNOS and TNFα (78, 106). Cinti et al. demonstrated that the differentiated M1 ATMs are exclusively localized in epididymal white adipose tissue (eWAT) and not inguinal white adipose tissue (iWAT), more specifically within “crown-like structures (CLS)” composed of necrotic adipocytes (107). The authors incorporated a HSL^−/−^ (hormone sensitive lipase KO) model to show that adipocyte hypertrophy itself triggers a pro-inflammatory microenvironment that facilitates infiltration of monocytes (107). The M1 monocyte-derived macrophages are recruited and stabilized by CCR2 and its ligand CCL2 in addition to other factors such as osteopontin and fatty acids (FAs) (78, 106). CCL2 loss of function as well as CCR2 gain of function or overexpression studies have collectively illustrated the fundamental role of CCR2 and CCL2 in recruitment and differentiation of macrophages with an M1 phenotype (78, 85). However, Amano et al. recently demonstrated that local proliferation of ATMs contributes significantly to tissue ATM accumulation. They further demonstrated that this *in situ* proliferation is driven by monocyte chemotactic protein (MCP-1) (108). These studies highlight the contribution of both local proliferation and infiltrating monocytes to the accumulation of ATMs in an obesity induced inflammatory state (108, 109).

While the observed ATM increase associated with obesity is predominantly attributed to macrophages exhibiting an M1 phenotype, Shaul et al. recently suggested that there exists an M1/M2 hybrid ATM population and that the increase in ATMs with obesity exhibit more of this mixed phenotype. The study noted an increase in both M1 and M2 gene transcripts in a mouse DIO model of chronic inflammation suggesting that perhaps both populations may influence the progression of obesity-associated chronic inflammation (103). However, despite transcriptional increases in both M1 and M2 genes, further studies by the authors demonstrated an overall decrease in the M2 populations in the DIO model as exhibited by flow cytometry analysis of eWAT cells expressing macrophage galactose type-C lectin-1 (MGL1, also known as CD301). It is not clear whether this intermediate population of ATMs represents a truly separate population of cells or whether these ATMs represent a transitional population, resulting from the inherent plasticity of ATM phenotypes *in vivo*.

In healthy adipose tissue, tissue remodeling is accompanied by angiogenesis to maintain oxygen supply and critical nutrients to promote adipose tissue homeostasis. However, under pathological conditions, the existing adipocytes enlarge, angiogenesis is limited, and tissue hypoxia ensues, resulting in the recruitment of inflammatory cells. Angiogenesis is induced by vascular endothelial factor-A (VEGF-A) stimulation of vascular endothelial growth factor receptor-2 (VEGFR-2) (110, 111). When VEGFR-2 is blocked early in the onset of obesity, the development of metabolic disorders is elevated suggesting that expanding vascularization can promote diet-induced disease (112). However, Elias et al. demonstrated that overexpression of VEGF-A in adipose tissue in a DIO model promotes the maintenance of a larger M2 population and attenuates adipose tissue inflammation (113), coinciding with an increase in local blood flow and decreased presence of necrotic CLS. The authors further conclude that the correlative increase in VEGF and M2 macrophage populations is likely due to recruitment of M2 macrophages to sites of inflammation in adipose tissue rather than promoting a phenotypic switch between M1 and M2 macrophage populations. Recent studies also demonstrate an increase in brown adipose tissue thermogenesis as a response to cold, particularly in the presence of VEGF overexpression (113, 114). Interestingly, pivotal studies in by the Chawla lab demonstrated that the absence of alternatively activated M2 macrophages results in impaired metabolic adaptation to cold, while administration of IL-4 promotes adaptation in a macrophage-dependent manner (115), suggesting that the increased thermogenesis in response to cold in the presence of VEGF could also be mediated, at least in part, by M2 macrophages.

Therapeutic strategies in obesity and obesity-associated disease may lie in understanding the molecular underpinnings of tissue-resident macrophage heterogeneity. For instance, loss of KLF4 function has been associated with a loss of wound healing capacities under inflammatory conditions suggesting an important role for KLF4 in maintaining an M2 phenotype *in vivo* (87). In addition to KLF4, differential expression of PPARδ and PPARγ in current experimental models, including HFD-induced obesity, illustrate a critical role for activated PPARs in directing monocytic infiltration and polarization of macrophages toward the alternative state in adipose tissue and liver (116, 117). Genetic deletion of PPARδ and/or PPARγ in macrophages directs the differentiation of monocytes toward a pro-inflammatory M1 phenotype (88, 118). Conversely, agonist mediated PPAR-activation stimulates monocytic differentiation into an alternately activated M2 phenotype (119). These agonists include synthetic anti-diabetic agents such as Thiazolidinediones (TZD) or natural agents such as abscisic acid (ABA) (116, 119, 120). Recent studies by Satoh et al. have demonstrated an essential role for the pseudokinase, Trib1, in the development of M2-like macrophage populations. Mice lacking Trib1 in the hematopoietic cell compartment exhibit a severe reduction in M2-like macrophages in a number of tissues under homeostatic conditions (121). In addition, these mice also exhibit a significant reduction in eosinophils, suggesting that the defect in M2 polarization could be, in part, secondary to diminished levels of eosinophil-derived IL-4 under homeostatic conditions. Importantly, these mice develop hypertriglyceridemia and insulin resistance when maintained on a HFD, associated with increased expression of pro-inflammatory cytokines (121, 122).

## Kupffer Cells (Liver-Resident Macrophages)

**Figure 4:**
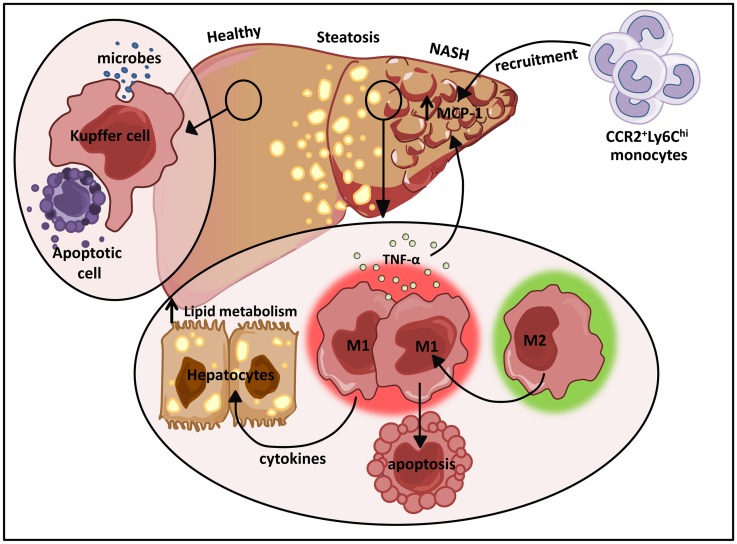
**The dual role of M1/M2 Kupffer cells in NAFLD**. Kupffer cells play a pivotal role in host defense where they routinely clear microbes and apoptotic bodies from portal circulation. During diet-induced liver injury, tissue-resident macrophages exhibiting a classically activated M1 phenotype predominate and secrete cytokines that can alter hepatocyte lipid metabolism and induce MCP-1 dependent recruitment of monocytes into the liver. In turn, these infiltrating cells facilitate the development and progression of NAFLD. Restrained induction of M1 Kupffer cell apoptosis further perpetuates liver inflammation.

One of the largest resident populations of macrophages exists in the liver. These YS macrophages, termed Kupffer cells, are positioned in the liver sinusoids where they efficiently clear microbes and apoptotic cells from the portal circulation (Figure 4) (123). While the proliferative capacity of hepatic macrophages remains controversial, recent studies have suggested that Kupffer cells are established prenatally and are maintained into adulthood independent of replenishment from blood monocytes in a manner dependent on M-CSF and GM-CSF (9). Kupffer cells also play essential roles in tissue homeostasis, tissue remodeling, and regulation of metabolic function in the liver (Figure 4). Due to their constant exposure to blood-borne food antigens and bacterial products of commensal intestinal flora, these cells are also required to prevent the onset of inflammation in response to these non-pathogenic stimuli (124). In contrast, monocyte-derived macrophages in the liver play dual roles in promoting both tissue inflammation and repair. These infiltrating cells are initially CD11b^+^F4/80^+^ and eventually differentiate into more mature CD11b^lo^F4/80^hi^ macrophages.

Following acute hepatic injury induced by acetaminophen, there is a large influx of monocyte-derived macrophages induced by CCL2, accompanied by a significant decrease in the resident population of Kupffer cells (125–127). This influx is blocked in CCR2-deficient mice and these mice exhibit a delay in tissue recovery indicating that the infiltrating population of macrophages plays an important role in promoting liver repair following an acute insult (126, 127). However, when both infiltrating hepatic macrophages and resident Kupffer cells are depleted, tissue recovery is dramatically impaired (126, 128). These mice exhibited impaired clearance of necrotic cells and a defect in the restoration of the liver microvasculature (126, 128). During the resolution phase of acute inflammation, the infiltrating monocyte-derived cells differentiate into the more mature reparative macrophages and the resident Kupffer cell population is restored. Genomic analysis of these co-existing populations of cells demonstrated that, while the recovered Kupffer cell population was similar to resident Kupffer cells before injury, these cells were distinct from the resolution phase monocyte-derived liver macrophage population, suggesting that these populations have distinct functions in liver recovery from acute inflammation and that the infiltrating monocyte-derived macrophages do not replace the resident Kupffer cells (126).

Non-alcoholic fatty liver disease is the most common liver disease in the western world, the prevalence of which has increased in parallel to the increased incidence of obesity (129, 130). This clinicopathological condition is characterized by lipid accumulation in hepatocytes and ranges from the non-progressive form termed steatosis to non-alcoholic steatohepatitis (NASH), the progressive form that is prone to the development of cirrhosis, liver cancer, and liver failure (130, 131). In response to obesity, Kupffer cells are engaged by cytokines, adipokines, and FAs secreted from adipose tissue promoting a polarized M1 phenotype (132). In addition, the release of FFAs into the liver by hypertrophic adipocytes leads to hepatocyte ER stress and ROS production (132). Hepatocyte death triggered by these responses results in the release of danger associated molecular patterns (DAMPs) that further promote liver macrophage polarization (123, 132).

In hepatic steatosis, pro-inflammatory cytokines produced by M1 activated macrophages induce hepatic cholesterologenesis and increase TAG production, resulting in discordant regulation of lipid metabolism and homeostasis (Figure 4) (133–136). Sterol regulatory element-binding proteins (SREBPs) directly activate genes that mediate the synthesis and uptake of cholesterol and FAs (137). Elevation of SREBPs often underlies the pathogenesis of insulin resistance and type 2 diabetes (138, 139). In addition to their initial propagation of fatty liver disease, activated M1 Kupffer cells trigger the more severe form of non-alcoholic fatty liver disease (NAFLD), NASH. A recent study demonstrated that TNFα produced by M1 hepatic macrophages increased intrahepatic expression of MCP-1 (Figure 4), a major chemokine responsible for CCR2-dependent recruitment of Ly6C^hi^ monocytes (135). The vast infiltration of Ly6C^hi^ monocytes further perturbs liver homeostasis amplifying hepatic inflammatory responses (Figure 4). The recruited monocyte-derived macrophage population also plays a key role in promoting fibrogenesis. In mice deficient for CCR2 or depleted of Kupffer cells, inflammatory cell infiltration, steatohepatitis, and fibrosis are ameliorated (135).

Progression of NAFLD to NASH occurs in a subset of patients with fatty liver disease, and this progression is largely linked to the activation of toll like receptor 4 (TLR4) by both exogenous and endogenous ligands. Bacteria and bacterial products, such as LPS, which translocate from the gut can activate TLR4 signaling to induce NASH (140, 141). Furthermore, endogenous high mobility group box-1 (HMGB-1) and FFAs have also been shown to be endogenous ligands for TLR4 (142, 143). A number of recent studies have also demonstrated a crucial link between the composition of gut microbiota, obesity, and the development of NASH (144). CD14 promotes the activation of TLR4 by promoting the recruitment of LPS to the TLR4 signaling complex (145). Interestingly, Imajo et al. demonstrated that the increased levels of leptin associated with obesity promotes the enhanced expression of CD14 in Kupffer cells, resulting in increased sensitivity of the Kupffer cells to low doses of LPS (146). Translocated bacterial DNA has also been shown to promote the development of NASH through engagement of TLR9 in Kupffer cells, which stimulates the production of interleukin-1β (IL-1β) (147). IL-1β in turn, promotes hepatocyte lipid accumulation and death, as well inducing hepatic stellate cells (HpSCs) to promote fibrosis.

Liver fibrosis is a common feature of chronic liver disease. It is characterized by accumulation of extracellular matrix (ECM) resulting from both increased synthesis and decreased degradation of ECM proteins (148). During progressive liver injury, HpSC-derived myofibroblast-like cells predominate and initiate collagen deposition (148). Extensive evidence demonstrates a complex interplay between Kupffer cells and HpSCs during hepatic fibrogenesis. In general, alternatively activated M2 macrophages are involved in tissue remodeling through the production of transforming growth factor beta-1 (TGFβ-1), a potent inducer of the HpSC fibrotic phenotype (149, 150). However, while arginase produced by M2 cells during a Th2 response was thought to play a role in progression of fibrosis, studies by Pesce et al., in which arginase is depleted in macrophages, highlight a role for arginase-expressing macrophages in the resolution of fibrosis (151). Alternatively, in hepatic fibrosis, Ly6C^hi^ inflammatory monocyte-derived macrophages express higher levels of TGFβ-1 and other pro-inflammatory cytokines that activate HpSCs (152). TGFβ-1 not only promotes the fibrogenic activity of HpSCs but also enhances expression of tissue inhibitors of metalloproteinases (TIMPs) by HpSCs that block the degradation of ECM. These inflammatory monocyte-derived macrophages also exhibit decreased expression of matrix metalloproteinases, thus directly promoting fibrosis.

Interestingly, a recent report demonstrated a role for a novel population of monocyte-derived macrophages in the resolution of fibrosis. These cells are derived from Ly6C^hi^ monocytes and exhibit a CD11b^hi^F4/80^int^Ly6C^lo^ phenotype. These cells mediate MMP-dependent degradation of the ECM and the expression profile of this population of resolving monocyte-derived macrophages demonstrates that these cells are distinct from the typical M1/M2 populations of macrophages (152). More recently, it has been reported that alternatively activated M2 Kupffer cells can promote caspase-3-dependent apoptosis of classically activated M1 Kupffer cells, thus providing a protective mechanism against NAFLD (Figure 4) (153). The M2 mediated apoptosis of M1 Kupffer cells was shown to be arginase dependent by way of IL-10 (153). Interestingly, alternatively activated M2 Kupffer cells limit hepatocyte apoptosis and steatosis in alcohol induced liver injury through the release of IL-6, a mechanism that may expand to NAFLD (154).

## Summary

**Figure 5:**
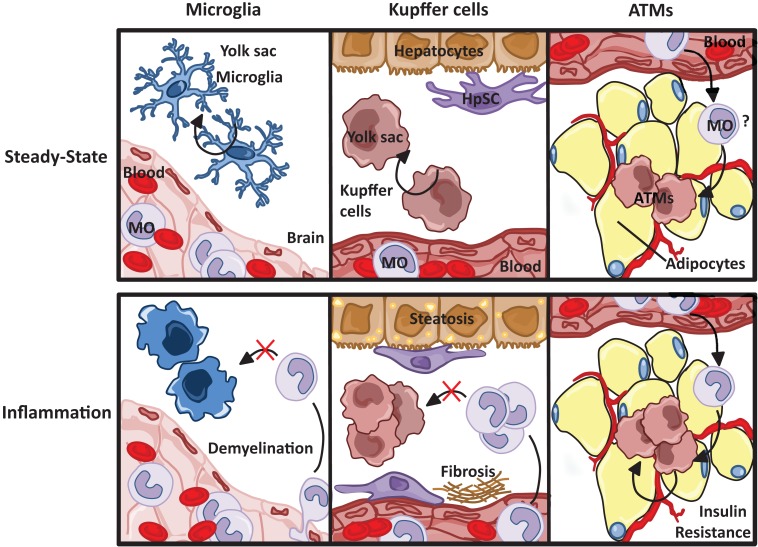
**Resident macrophage populations in steady state and inflammation**. Resident microglia and Kupffer cell populations, both yolk sac-derived, are maintained through self-renewal, unlike macrophages resident to the adipose tissue, which are thought to originate from circulating blood monocytes (MO). Although the progression of tissue specific pathophysiological insults are dependent on both resident and infiltrating macrophages, monocyte-derived macrophages do not replenish resident microglia and Kupffer cell populations.

Based on recent studies, a unifying theme is beginning to emerge between the contribution of tissue-resident macrophages and infiltrating monocyte-derived macrophages in chronic inflammation (Figure 5). (1) Both microglia and Kupffer cells are of embryonic origin. Microglia are YS, while contribution of yolk sac and fetal liver has been described for Kupffer cells. However, because it now appears that the yolk sac progenitors seed the liver where they continue to contribute to tissue-resident macrophage populations independent of hematopoietic stem cells, it is likely that these cells share a common progenitor and are ontologically indistinguishable. (2) In the brain and liver, resident macrophages and infiltrating monocyte-derived macrophages play distinct roles in the progression of inflammation. In the brain, microglia are required for the initiation of disease, while infiltrating monocyte-derived macrophages promote demyelination. In the liver, both resident and infiltrating populations contribute to both inflammation and repair, Kupffer cells are largely responsible for the development of fibrosis. (3) In the brain and liver, infiltrating monocytes do not contribute to the resident macrophage pool rather, during recovery, resident cells are replenished through local proliferation. (4) In both the brain and liver, there is evidence that the resident population of macrophages plays a role in promoting apoptosis and/or clearance of the infiltrating monocyte-derived macrophage population during the recovery phase.

However, the relative contribution of resident versus infiltrating populations of macrophages in adipose tissue remains obscure. While ATMs are generally considered to be hematopoietic stem cells-derived, this assumption is based on studies using myeloablation. Further fate mapping studies will be needed to definitively identify the origin of ATMs. In addition, while it is clear that both infiltration of monocytes and proliferation of resident macrophage contribute to the massive expansion of ATMs in obesity, the respective roles of resident versus infiltrating macrophages in the propagation and/or resolution of inflammation is unclear. Finally, it unknown whether infiltrating monocyte-derived macrophages in adipose tissue ultimately contribute to the resident population.

In all cases, the M1/M2 polarization of macrophages in inflammation and disease resolution has been described. However, these are largely based on studies that do not distinguish between resident macrophage and infiltrating macrophage populations. Given the emergence of technologies that help to distinguish resident cells from infiltrating cells, it will be essential to re-visit the M1/M2 paradigm in the context of macrophage ontogeny to determine the extent of plasticity of individual populations of macrophages.

The recognition that the M1/M2 polarization of macrophages plays a central role in the progression of chronic inflammation in a wide range of diseases and that therapeutic approaches to chronic inflammatory disease could involve the regulation of macrophage polarization renders these outstanding questions highly significant. Taking into consideration not only the polarization state of macrophages, but also their ontogeny and phenotypic plasticity, will be central in the development of therapeutic approaches for the treatment of chronic inflammatory disease aimed at redirecting macrophage polarization and function.

## Conflict of Interest Statement

The authors declare that the research was conducted in the absence of any commercial or financial relationships that could be construed as a potential conflict of interest.
